# Effects of water stress on starch synthesis and accumulation of two rice cultivars at different growth stages

**DOI:** 10.3389/fpls.2023.1133524

**Published:** 2023-04-25

**Authors:** Guangyi Chen, Ligong Peng, Jing Gong, Jin Wang, Chaoyue Wu, Xiaodong Sui, Yunfeng Tian, Mingming Hu, Congmei Li, Xingmei He, Hong Yang, Qiuqiu Zhang, Yuyuan Ouyang, Yan Lan, Tian Li

**Affiliations:** ^1^ College of Agronomy, Sichuan Agricultural University, Chengdu, China; ^2^ College of Agronomy, South China Agricultural University, Guangzhou, China; ^3^ Rice Research Institute, Sichuan Agricultural University, Chengdu, China; ^4^ Crop Research Institute, Sichuan Academy of Agricultural Sciences, Chengdu, China; ^5^ College of Life Science and Engineering, Southwest University of Science and Technology, Mianyang, China

**Keywords:** rice, water stress, starch synthesis, enzyme activity, yield

## Abstract

Rice is a water intensive crop and soil water conditions affect rice yield and quality. However, there is limited research on the starch synthesis and accumulation of rice under different soil water conditions at different growth stages. Thus, a pot experiment was conducted to explore the effects of IR72 (indica) and Nanjing (NJ) 9108 (japonica) rice cultivars under flood-irrigated treatment (CK, 0 kPa), light water stress treatment (L, -20 ± 5 kPa), moderate water stress treatment (M, -40 ± 5 kPa) and severe water stress treatment (S, -60 ± 5 kPa) on the starch synthesis and accumulation and rice yield at booting stage (T1), flowering stage (T2) and filling stage (T3), respectively. Under LT treatment, the total soluble sugar and sucrose contents of both cultivars decreased while the amylose and total starch contents increased. Starch synthesis-related enzyme activities and their peak activities at mid-late growth stage increased as well. However, applying MT and ST treatments produced the opposite effects. The 1000-grain weight of both cultivars increased under LT treatment while the seed setting rate increased only under LT3 treatment. Compared with CK, water stress at booting stage decreased grain yield. The principal component analysis (PCA) showed that LT3 got the highest comprehensive score while ST1 got lowest for both cultivars. Furthermore, the comprehensive score of both cultivars under the same water stress treatment followed the trend of T3 > T2 > T1, and NJ 9108 had a better drought-resistant ability than IR72. Compared with CK, the grain yield under LT3 increased by 11.59% for IR72 and 16.01% for NJ 9108, respectively. Overall, these results suggested that light water stress at filling stage could be an effective method to enhance starch synthesis-related enzyme activities, promote starch synthesis and accumulation and increase grain yield.

## 1 Introduction

Rice (*Oryza sativa* L.) is one of the main food crops consumed worldwide and provides about 35% of the dietary calorie intake for more than 3 billion people (Fageria, 2011). Rice is also the largest consumer of water and water management will have important impacts on its yield and quality (Bam et al., 2007). China is the main producer of rice with planting area and production accounting for 23% and 30% of the world total, respectively (Wei et al., 2020). At present, traditional continuous flooding irrigation is the major rice production system which consuming nearly 70% of the irrigated fresh water resources in China (Yao et al., 2014; Wang, 2021). However, China is one of the 13 countries with water shortage which has only 8% of the world’s available fresh water resources (Zhou, 2013). Water deficit is a serious environmental stress and the major constraint to rice production (Rehmani et al., 2014). Losses in rice yield due to water shortage probably exceed losses from all other causes combined and the extent of the yield loss depends on both the severity and duration of the water stress (Wu et al., 2014). In recent years, the water deficit problems are likely to worsen in the future with predicted climate change scenarios (Passioura, 2007; Cao et al., 2017). Due to the high temperature, the uneven spatial and temporal distribution of rainfall during rice large water requirement period (July and August) (Wang et al., 2013; Wang et al., 2015b), water stress has become the major challenge limiting rice production in Sichuan (Pandey and Shukla, 2015). Therefore, how to improve water use efficiency and optimize rice water management without decreasing rice yield and quality have always been hot research topics and are also of great significance to food security and social stability (Liu et al., 2014a).

Rice quality is mainly determined by starch which accounts for 80% of the total mass of rice grains. Sucrose is the initial substance and starch is the final product during rice grain carbohydrate metabolism. As starch in rice endosperm contributes 90% of the final dry weight of an unpolished grain, the rice grain filling is actually a process of sucrose conversion and starch accumulation which may have direct impact on rice yield and quality (Li et al., 2018). It has been reported that there are over 30 enzymes involved in starch synthesis. Among them, five enzymes are considered to play key roles in this process, which are adenosine diphosphate-glucose pyrophosphorylase (AGPase), granule bound starch synthetase (GBSS), soluble starch synthase (SSS), starch branching enzyme (SBE) and starch debranching enzyme (DBE) (Chen et al., 2021). The activities of these five enzymes are closely related to total starch, amylose and amylopectin accumulation in rice endosperm. Studies have shown that appropriate water stress (re-watered when soil water potential reached at -15 kPa) could enhance the activities of the key enzymes involved in the conversion from sucrose to starch and promote the translocation and redistribution of reserved carbohydrates in vegetative organs to grain yield (Cai et al., 2008; Xu et al., 2018). A moderate dry-wet alternate irrigation (re-watered when soil water potential reached at -25 kPa) during the grain filling stage increased the activities of AGPase, SBE, sucrose synthase (SuS) and starch synthase (StS) and improve rice quality while the results were reversed for the severe dry-wet alternate irrigation (re-watered when soil water potential reached at -50 kPa) (Yang et al., 2005). Both moderate soil-drying (soil water potential at -10~-30 kPa) and alternate wetting and moderate-drying irrigation (re-watered when soil water potential reached at -25 kPa) could improve rice quality, resulting from the physiological mechanism of enhancing activities of AGPase, SBE, SuS, and StS and decreasing ethylene production in grains (Liu et al., 2008; Liu et al., 2014b).

Although many studies have investigated the effects of water stress on rice water use efficiency, water-requiring property, plant type, stomatal characteristics, leaf photosynthesis and transpiration characteristics, root morphology and physiology, water absorption and transportation, plant hormones and so on. However, conclusions differed due to the different ecological conditions, cultivars, water stress severity and duration (Zhang et al., 2008). There is still limited information on the dynamic changes of starch accumulation and related enzyme activities under different soil water conditions before rice maturity stage. A better understanding of physiological and biochemical changes at different growth stages of rice will be helpful in choosing appropriate water management to achieve high yield and quality. Thus, we conducted a pot experiment to explore the effects of flood-irrigated condition, light water stress condition, moderate water stress condition and severe water stress condition on starch synthesis and accumulation and rice yield at booting stage, flowering stage and filling stage, respectively. The present study was aimed to provide a theoretical basis for high-yield and water-saving cultivation of rice.

## 2 Materials and methods

### 2.1 Experimental site and materials

**Figure 1:**
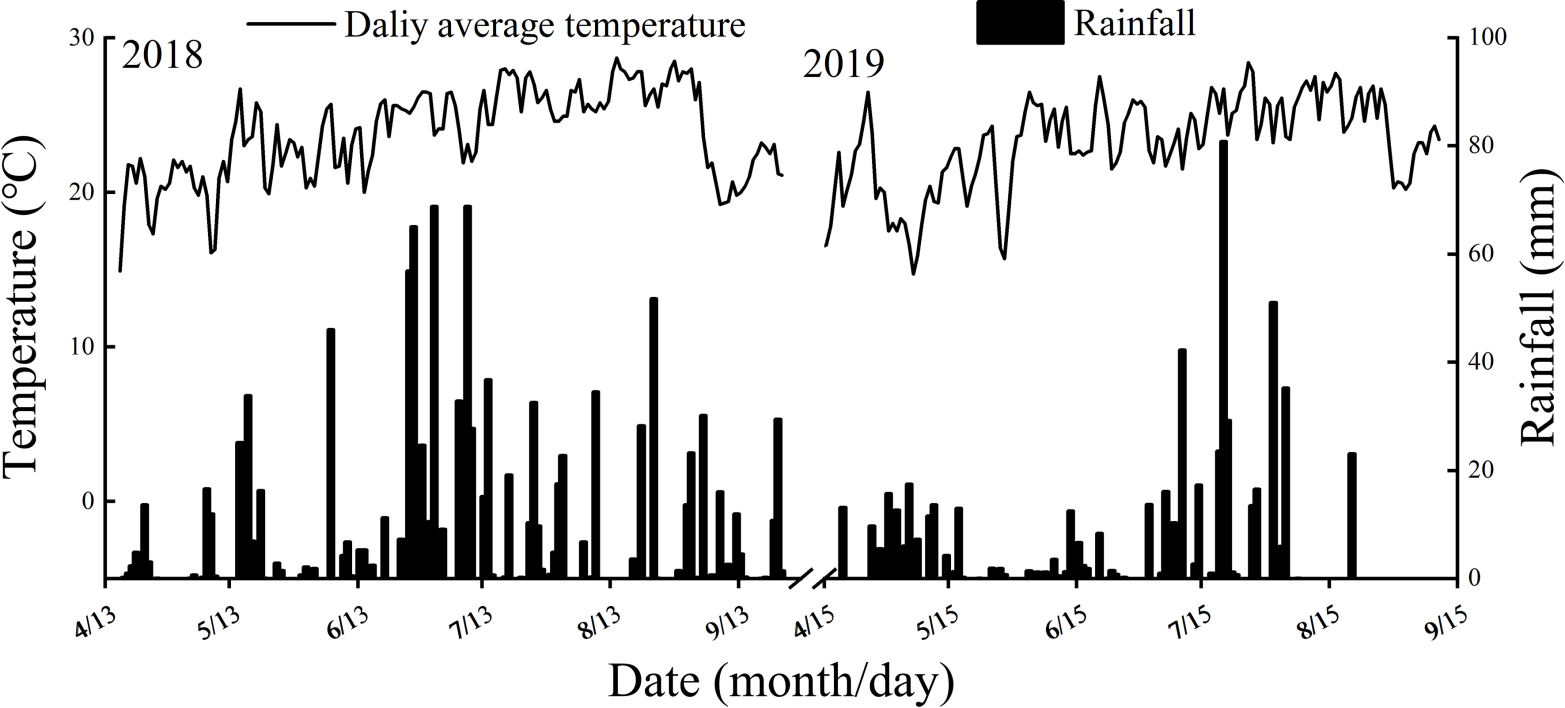
Climate data during the experimental periods.

Pot experiments were conducted during the growing seasons of 2018 and 2019 at the research farm of Sichuan Agricultural University, Wenjiang city, Sichuan Province, China (30°43′ N, 103°47′ E). The soil of the plot was clay soil. Prior to the establishment of the pot experiment, soil samples from the topsoil layer (0.20 m) were analyzed. The climate data and analysis results of the top soil layer were shown in **Figure 1** and **Table 1**, respectively. Two rice cultivars with significant differences in total starch content were used as the test materials. NJ 9108 (Jiangsu Academy of Agricultural Sciences, the whole growth period is about 153 days) is a japonica rice cultivar and IR72 (International Rice Research Institute, the whole growth period is about 133 days) is an indica rice cultivar, both with high yield and good quality.

**Table 1:** Soil properties of the top soil layer (0.20 m) at the experimental sites.

Years	pH	Organic matter (g kg^-1^)	Total N (g kg^-1^)	Total P (g kg^-1^)	Total K (g kg^-1^)	Available N (mg kg^-1^)	Available P (mg kg^-1^)	Available K (mg kg^-1^)
2018	5.83	30.95	1.95	0.751	7.32	100.75	25.91	56.52
2019	5.91	29.50	1.81	0.786	7.17	97.64	23.04	58.31

N, P, K represent nitrogen, phosphorus and potassium, respectively.

### 2.2 Experimental design

A randomized block design with 30 replicates per treatment was employed at booting stage (T1, 30% of rice enter the panicle differentiation stage), flowering stage (T2, 30% of rice begin to flower) and filling stage (T3, 30% of rice enter the milk stage), respectively. Four water treatments were assigned: flood-irrigated treatment (CK, 0 kPa), light water stress treatment (L, -20 ± 5 kPa), moderate water stress treatment (M, -40 ± 5 kPa) and severe water stress treatment (S, -60 ± 5 kPa), respectively. The water treatments were 10 days at each growth stage. And after the water treatments, plants were re-watered to permit recovery. A 2.5-meter-high rain shelter consisting of a steel frame covered with transparent film (the top to the bottom was not closed, and the transparent film was mounted approximately 0.5-0.6 m above the plant canopy to ensure ventilation) was built in each treatment to avoid the effect of rainfall precipitation on the treatments, and was removed after the treatment.

### 2.3 Field management and plant cultivation

Plastic pots (27 cm in height, 22 cm in bottom inside diameter, and 30 cm in top inner diameter) were filled with 12 kg soil where 2.16 g N, 1.08 g P_2_O_5_, and 2.16 g K_2_O fertilizers as urea, calcium superphosphate, and potassium chloride, respectively, were mixed. Before pot filling, the soil was kept under shade and air-dried, crushed and passed through a 2-mm sieve. N fertilizer was used as basal manure and top dressing at a 3:7 ratio. Basal N, P and K were applied to the soil 1 day (d) before transplanting. Seeds were sown on 17 April 2018 and 14 April 2019, and the seedlings were transplanted on 27 May 2018 and 24 May 2019, respectively. The seedlings were transplanted into each pot with two hills per pot and two seedlings per hill. Every pot was flooded with tap water to maintain 1-3 cm water layer except during the treatment application periods. The pots were regularly hand weeded, and insecticides were applied to control insect pests.

### 2.4 Water measurements

**Figure 2:**
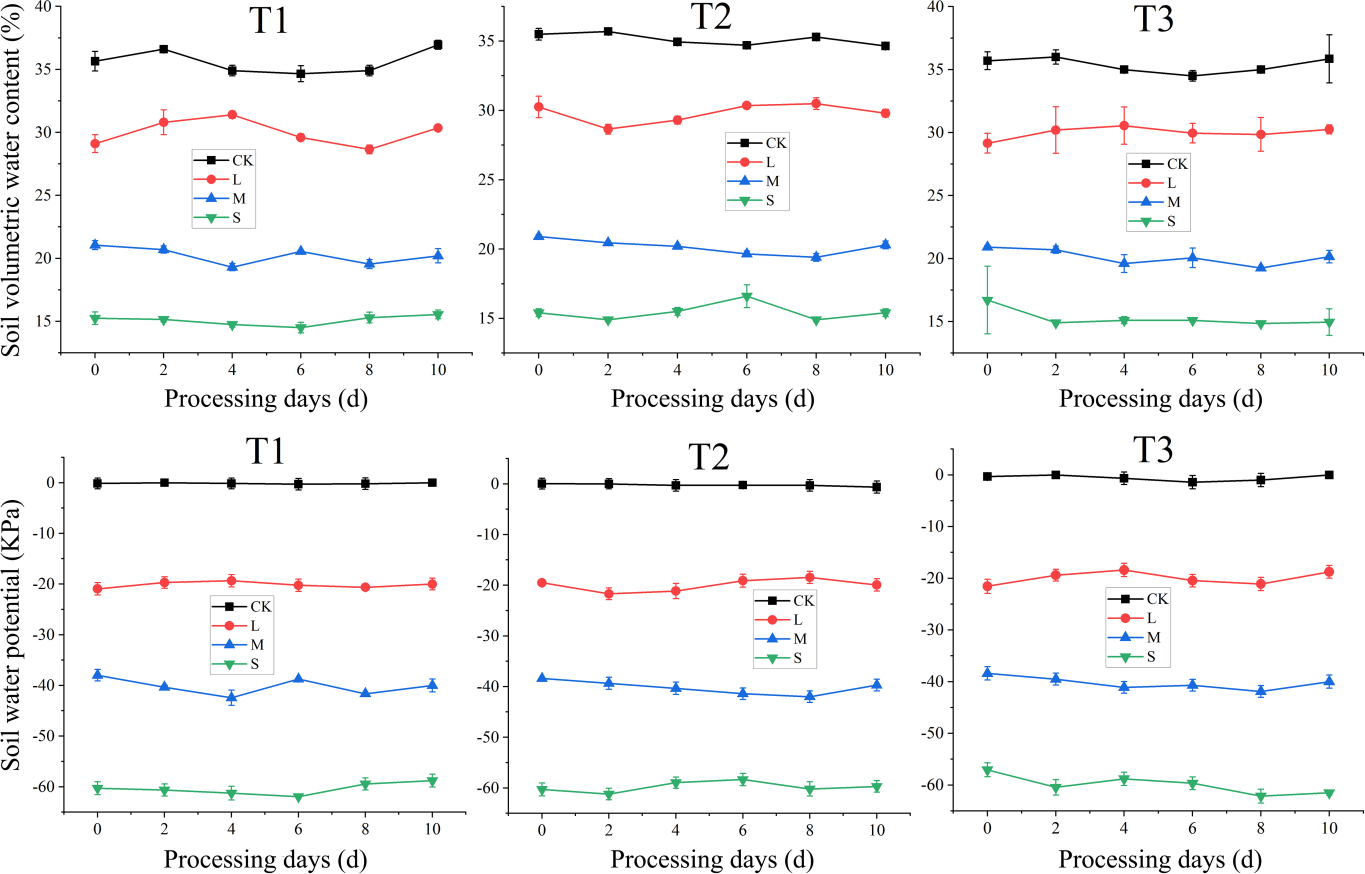
Soil water potential and soil volumetric water content during the treatment application periods. T1, T2, and T3 refer to the different growth stages (booting stage, flowering stage and filling stage, respectively). CK, L, M and S refer to the different water treatments (flood-irrigated treatment, 0 kPa, light water stress treatment, -20 ± 5 kPa, moderate water stress treatment, -40 ± 5 kPa and severe water stress treatment, -60 ± 5 kPa, respectively).

To record soil water potential, a soil moisture tensiometer (2725 ARL, Soil moisture Equipment Corp., Santa Barbara, CA, USA) with sensors immersed in below 10 cm of soil layer was used. Soil water content in pots was measured by a soil moisture sensors (EM 50, Decagon, Pullman, WA, USA). The soil moisture sensors were set with the tips of sensors at the middle point between plant and pot border, 5 cm below the soil surface. Sensors measured the dielectric constant of bulk soil and then converted these data to the values of volumetric water content. The recording interval time was 30 min, and then raw recorded data were averaged for each day. Average values of soil water potential and soil volumetric water content during the treatment application periods are shown in **Figure 2**.

### 2.5 Experimental conditions and procedures

#### 2.5.1 Preparation for determination

After flowering, approximately 200 panicles were selected on the same day and tagged for each treatment. After full heading, 30 tagged panicles were sampled from each treatment every 6 d at 10:00 a.m. The collected panicles were divided into two groups. Twenty tagged panicles were dried at 80°C, after which the brown rice was crushed and sieved through a 100-mesh screen for measurements of sucrose, total soluble sugar, amylose and total starch contents. Another 10 tagged panicles were placed in liquid nitrogen for 3 min and then stored at -80°C for enzymatic analysis and RNA extraction. At harvest, 6 pots from each treatment were sampled randomly and allowed to dry naturally in the sun to assess grain yield after the material was stored at room temperature for 3 months.

#### 2.5.2 Sucrose, total soluble sugar, amylose and total starch contents

The contents of sucrose, total soluble sugar and total starch (mg g^-1^ of dry brown rice weight) were measured by the anthrone colorimetric method (Gao, 2006). 0.1 g rice flour sample was extracted by 5.0 mL 80% ethanol at 80°C for 30 min. After repeated extraction and centrifugation (6000 r min^-1^ for 5 min) for three times, the supernatant (testing solution) was combined and the volume was adjusted to 100 mL. Aliquots (2 mL) of the extract were analyzed for sucrose and total soluble sugar content. The remaining precipitate was used for the determination of total starch content.

The amylose content (mg g^-1^ of dry brown rice weight) was measured by the iodine reagent method (Chen et al., 2020). 10 mL 0.5 mol L^-1^ KOH was added to 1.0 g rice flour sample, followed by the addition of 5.0 mL 1.0 mol L^-1^ HCl and 0.5 mL iodine reagent. After adjustment to 100 mL with distilled water, the absorbance was measured at 620 nm after 20 min by scanning the iodine absorption spectrum from 400 to 900 nm with a spectrophotometer (Ultrospec 6300 pro, Amershan Biosciences, Cambridge, Sweden). The values were converted to amylose content by reference to a standard curve prepared from rice.

#### 2.5.3 Activities of starch synthesis enzymes

The activities of AGPase, GBSS, SSS, SBE and DBE were measured by using ELISA Kits (Shanghai Fankel Industrial Co., Ltd., Shanghai, China). One unit of enzyme activity was defined as the amount that causes one unit absorbance increment per g of fresh weight per min. Based on the double antibody sandwich method, the optical density of samples was measured at 450 nm by using a microplate reader (Multiskan SkyHigh, Thermo Fisher Scientific, Waltham, MA, USA), and then the concentration of enzyme activity in the sample were calculated according to the standard curve.

#### 2.5.4 RNA isolation and RT-qPCR

Total RNA samples were obtained from rice grains at different growth stages (12 days after flowering for IR72 and 18 days after flowering for NJ 9108) using RNA Trizol reagent (Invitrogen, Carlsbad, CA, USA) and reverse-transcribed into cDNA using Revertase Transcription kit (Nanjing Vazyme Medical Technology Co., Ltd., Nanjing, China). The products were quantified using a real-time PCR detection system, following the manufacturer’s instructions (SYBR Green Master Mix, Vazyme). The rice Actin gene was used as an internal control. The PCR primers used were listed in **Supplementary Table 1**.

#### 2.5.5 Yield and yield components

Rice was harvested at maturity stage and the yield in each treatment was recorded after measuring moisture content and removing impurities. Grain yield was adjusted to a moisture content of 14%. The number of effective tillers per hill was determined before harvest using 6 pots per treatment. A total of 24 selected plants were separated into single tillers according to the marked date and were used to measure productive panicle number per pot, filled grain number per panicle, 1000-grain weight, seed setting rate and grain yield per pot.

### 2.6 Statistical analysis

An analysis of variance (ANOVA) model was performed to test the effects of the water stress on starch accumulation, starch synthesis-related enzyme activities and rice yield. And principal component analysis (PCA) of 10 indexes including starch synthesis-related enzyme activities, amylose and total starch content, seed setting rate, 1000-grain weight and yield of two cultivars among different water treatments was used to establish a comprehensive evaluation model. For the analysis, year, cultivar, water treatment and sampling time were considered fixed effects, whereas the replicates were considered random effects. The means of each treatment were compared based on the least significant difference (LSD) test at the 0.05 probability level by using SPSS 20.0 (Statistical Product and Service Solutions Inc., Chicago, IL, USA). Origin Pro 2020 (OriginLab, Northampton, MA, USA) was used to draw the figures. The differences of the main indicators are shown in **Table 2**. Variance analysis showed that the results of key enzyme activities during starch synthesis showed the same trend in both 2018 and 2019. Therefore, we showed the results in 2018 at further results sections.

**Table 2:** Analysis of variance on starch contents, starch synthesis-related enzyme activities and yield of NJ 9108 and IR72.

ANOVA	Year (Y)	Cultivar (C)	Treatment (T)	Y×C	Y×T	C×T	Y×C×T
SC	48.71**	6247.63**	161.01**	5.20*	0.58ns	55.03**	1.31ns
SSC	2.5ns	2260.10**	84.11**	1.10ns	1.88ns	2.23*	0.68ns
AC	42.33**	67498.18**	20.33**	10.18**	0.88ns	1.00ns	0.59ns
TSC	56.11**	1063.13**	102.96**	9.41**	1.03**	0.28ns	0.68ns
AGPase	163.77**	1552.38**	166.07**	0.74ns	52.54**	7.19**	21.13**
GBSS	1.72ns	8.40**	75.83**	9.02**	8.98**	0.29ns	4.85*
SSS	8.43**	58.29**	736.88**	40.88**	65.31*	0.29ns	1.65ns
SBE	0.11ns	49.92**	600.05**	36.06**	15.24**	2.65*	8.65**
DBE	0.40ns	184.42**	2993.43**	56.22**	6.84*	0.50ns	1.32ns
PPN	3.65ns	736.85**	2.26*	24.39**	1.56ns	0.62ns	2.18*
FGN	263.58**	2909.81**	17.52**	89.81**	22.03**	2.18*	0.49ns
SSR	294.10**	146.54**	348.88**	1.15ns	13.86**	11.98**	9.60**
1000-GW	6857.71**	50802.25**	3039.97**	392.25**	119.32**	81.19**	96.33**
GY	134.08**	522.41**	87.35**	510.91**	1.96ns	2.23*	3.38**

SC, SSC, AC, TSC, PPN, FGN, SSR, 1000-GW and GY represent the sucrose content, total soluble sugar content, amylose content, total starch content, productive panicle number per pot, filled grain number per panicle, seed setting rate, 1000-grain weight and grain yield per pot, respectively. ANOVA P values and symbols were defined as: *P<0.05; **P<0.01; ns, P>0.05.

## 3 Results

### 3.1 Starch synthesis and accumulation

#### 3.1.1 Total soluble sugar and sucrose contents

**Figure 3:**
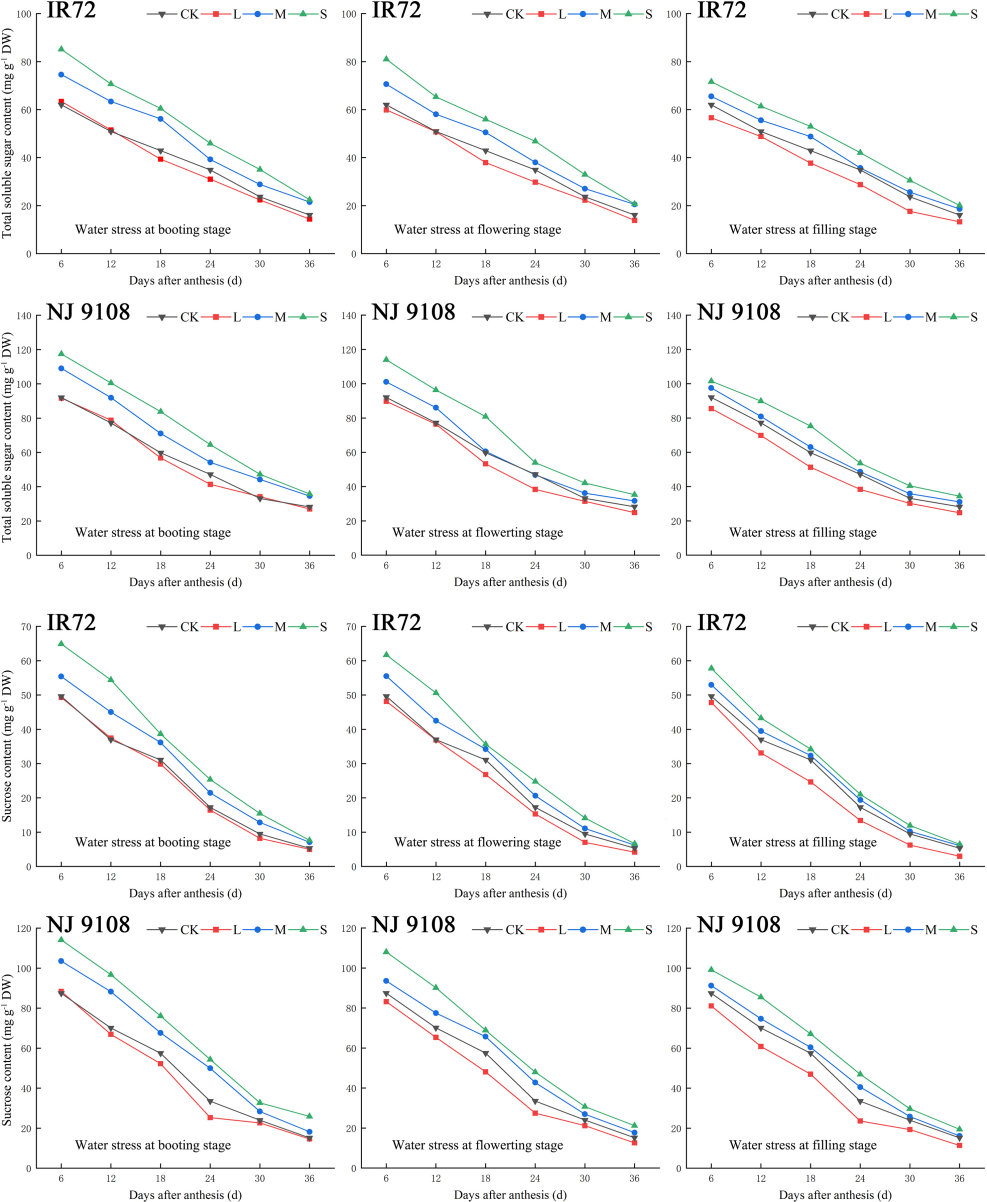
Effects of different water stress treatments on accumulation of total soluble sugar and sucrose in rice grains at different growth stages. T1, T2, and T3 refer to the different growth stages (booting stage, flowering stage and filling stage, respectively). CK, L, M and S refer to the different water treatments (flood-irrigated treatment, 0 kPa, light water stress treatment, -20 ± 5 kPa, moderate water stress treatment, -40 ± 5 kPa and severe water stress treatment, -60 ± 5 kPa, respectively). DW represents dry weight. The data presented are the average of two-year test.

During the grain filling process, the contents of total soluble sugar and sucrose in rice grains of both two cultivars showed a tendency to decrease gradually (**Figure 3**). As the water stress increased, the contents of total soluble sugar and sucrose of both two cultivars under the same growth stage increased significantly and tended to be in the order of S > M > CK > L (**Table 3**).

**Table 3:** Effects of different water stress treatments on total soluble sugar and sucrose contents in rice grains at maturity stage (mg g^-1^).

Year	Growth stage	Treatment	Total soluble sugar content		Sucrose content	
			IR72	NJ 9108	IR72	NJ 9108
2018	Booting stage	LT1	14.30 ± 1.80cd	27.34 ± 1.31ef	4.66 ± 0.58c	14.30 ± 0.39e
		MT1	20.80 ± 0.90ab	33.04 ± 1.72abc	6.37 ± 1.26ab	17.24 ± 0.58cd
		ST1	21.90 ± 0.25a	35.11 ± 1.78a	7.30 ± 0.62a	26.72 ± 0.81a
	Flowering stage	LT2	13.57 ± 0.69d	24.34 ± 2.60f	3.85 ± 0.59c	12.26 ± 0.94f
		MT2	19.56 ± 0.49b	31.03 ± 1.40cd	6.23 ± 0.21b	16.14 ± 0.55de
		ST2	20.22 ± 2.53ab	34.67 ± 1.60ab	6.29 ± 0.64ab	20.39 ± 1.86b
	Filling stage	LT3	13.04 ± 1.12d	26.08 ± 2.28ef	2.64 ± 0.34d	11.05 ± 0.55f
		MT3	19.14 ± 1.27b	31.72 ± 0.82bc	5.91 ± 0.26b	15.24 ± 0.78e
		ST3	19.70 ± 0.35b	35.07 ± 0.48a	6.14 ± 0.68b	18.78 ± 2.29bc
		CK	15.87 ± 0.09c	28.52 ± 2.79de	4.73 ± 0.39c	14.94 ± 0.80e
2019	Booting stage	LT1	14.49 ± 2.01de	26.67 ± 0.18de	5.32 ± 0.01d	14.79 ± 0.79e
		MT1	22.19 ± 1.48ab	36.12 ± 2.04a	7.75 ± 0.48a	19.13 ± 0.45c
		ST1	23.10 ± 1.09a	36.44 ± 0.79a	7.96 ± 0.11a	28.05 ± 0.81a
	Flowering stage	LT2	14.17 ± 0.69de	25.34 ± 2.07ef	4.52 ± 0.57e	12.93 ± 0.31f
		MT2	21.55 ± 0.72ab	32.28 ± 1.27bc	6.24 ± 0.51bc	19.30 ± 0.48c
		ST2	21.09 ± 2.81ab	35.94 ± 0.82a	6.95 ± 0.22b	22.96 ± 1.29b
	Filling stage	LT3	13.50 ± 1.12e	23.41 ± 1.14f	3.30 ± 0.35f	11.72 ± 1.13f
		MT3	18.11 ± 1.23c	30.43 ± 1.52c	6.24 ± 0.48bc	17.04 ± 0.34d
		ST3	20.57 ± 1.25b	33.74 ± 0.93b	6.80 ± 0.49b	20.11 ± 0.61c
		CK	16.30 ± 0.02cd	27.97 ± 0.16d	5.92 ± 0.77cd	15.39 ± 1.55e

T1, T2, and T3 refer to the different growth stages (booting stage, flowering stage and filling stage, respectively). CK, L, M and S refer to the different water treatments (flood-irrigated treatment, 0 kPa, light water stress treatment, -20 ± 5 kPa, moderate water stress treatment, -40 ± 5 kPa and severe water stress treatment, -60 ± 5 kPa, respectively). Lower case letters indicate that the contents of total soluble sugar and sucrose of both cultivars are significantly different with the different treatments in the same column (P<0.05, LSD method). The data presented are the mean ± standard deviation, n = 3.

The highest total soluble sugar contents of both two cultivars were observed under ST1 while the lowest values were observed under LT3 (IR72) and LT2 (NJ 9108), respectively, in 2018. The two-year test results showed the same trend except that the lowest total soluble sugar content of NJ 9108 was observed under LT3 in 2019. No significant differences were found between LT1, LT2 and LT3. Compared with that under CK, the total soluble sugar content of IR72 decreased by 9.89%, 14.50% and 17.83%, respectively, under LT1, LT2 and LT3, and increased by 38.00%, 27.41% and 24.13%, respectively, under ST1, ST2 and ST3. The total soluble sugar content of NJ 9108 decreased by 4.14%, 14.66% and 8.56%, respectively, under LT1, LT2 and LT3, and increased by 23.11%, 21.56% and 22.97%, respectively, under ST1, ST2 and ST3 when compared with that under CK.

The highest sucrose contents of both two cultivars were observed under ST1 while the lowest values were observed under LT3 in 2018. The two-year test results showed the same trend. Significant difference was found between LT3 and LT1. Compared with that under CK, the sucrose content of IR72 decreased by 1.48%, 18.60% and 44.19%, respectively, under LT1, LT2 and LT3, and increased by 54.33%, 32.98% and 29.81%, respectively, under ST1, ST2 and ST3. The sucrose content of NJ 9108 decreased by 4.28%, 17.94% and 26.04%, respectively, under LT1, LT2 and LT3, and increased by 78.85%, 36.48% and 25.70%, respectively, under ST1, ST2 and ST3 when compared with that under CK.

Taken together, these results indicated that light water stress could decrease the contents of total soluble sugar and sucrose in rice grains of which the effect at filling stage was more obvious. On the contrary, moderate and severe water stress could increase the contents of total soluble sugar and sucrose of which the effect at booting stage was more obvious.

#### 3.1.2 Aymlose and total starch contents

**Figure 4:**
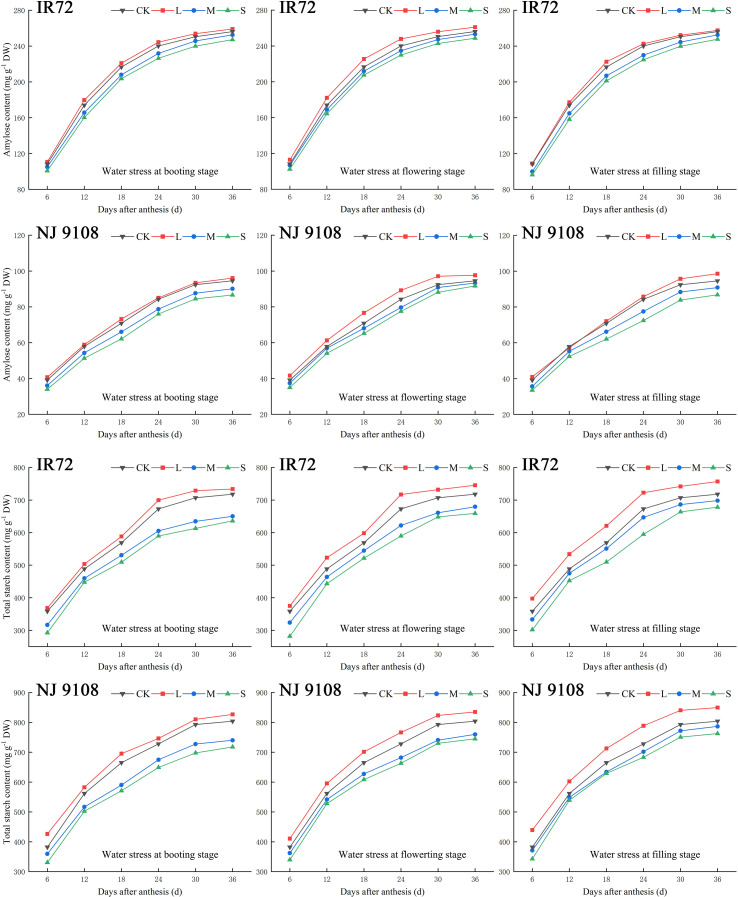
Effects of different water stress treatments on accumulation of amylose and total starch in rice grains at different growth stages. T1, T2, and T3 refer to the different growth stages (booting stage, flowering stage and filling stage, respectively). CK, L, M and S refer to the different water treatments (flood-irrigated treatment, 0 kPa, light water stress treatment, -20 ± 5 kPa, moderate water stress treatment, -40 ± 5 kPa and severe water stress treatment, -60 ± 5 kPa, respectively). DW represents dry weight. The data presented are the average of two-year test.

During the grain filling process, the contents of amylose and total starch in rice grains of both two cultivars increased gradually and then stabilized (**Figure 4**). As the water stress increased, the contents of amylose and total starch of both two cultivars under the same growth stage decreased significantly and tended to be in the order of L > CK > M > S (**Table 4**).

**Table 4:** Effects of different water stress treatments on amylose and total starch contents in rice grains at maturity stage (mg g^-1^).

Year	Growth stage	Treatment	Amylose content		Total starch content	
			IR72	NJ 9108	IR72	NJ 9108
2018	Booting stage	LT1	261.57 ± 0.27ab	94.82 ± 0.42abc	844.9 ± 12.04a	739.0 ± 6.93ab
		MT1	256.19 ± 0.49cde	93.21 ± 0.09bcd	757.2 ± 20.17c	656.3 ± 7.87de
		ST1	249.87 ± 0.26f	89.49 ± 0.32d	724.9 ± 23.34d	639.6 ± 12.88e
	Flowering stage	LT2	264.01 ± 0.19a	98.40 ± 0.46a	846.1 ± 5.41a	749.3 ± 14.92ab
		MT2	258.05 ± 0.28bcd	95.07 ± 0.12abc	777.7 ± 25.68c	692.0 ± 7.83c
		ST2	252.38 ± 0.33ef	91.33 ± 0.12cd	755.4 ± 15.25c	666.1 ± 21.85d
	Filling stage	LT3	260.17 ± 0.04abc	97.82 ± 0.44ab	850.5 ± 12.61a	756.8 ± 16.72a
		MT3	254.78 ± 0.35def	92.04 ± 0.34cd	811.2 ± 22.14b	698.9 ± 8.47c
		ST3	249.87 ± 0.20f	88.12 ± 0.19d	770.5 ± 14.57c	688.1 ± 10.92c
		CK	258.65 ± 0.25bcd	96.05 ± 0.16abc	822.2 ± 6.03ab	730.9 ± 4.95b
2019	Booting stage	LT1	256.64 ± 0.59ab	97.30 ± 0.14ab	808.8 ± 19.07bc	728.7 ± 10.19b
		MT1	248.78 ± 0.15cd	86.95 ± 0.38efg	722.8 ± 7.99gh	644.0 ± 8.70e
		ST1	244.58 ± 0.27d	83.75 ± 0.14g	710.9 ± 7.03h	639.6 ± 12.88e
	Flowering stage	LT2	258.04 ± 0.56a	96.87 ± 0.26abc	823.7 ± 7.48ab	741.3 ± 8.00ab
		MT2	248.26 ± 0.54cd	91.53 ± 0.38de	741.9 ± 25.62efg	666.7 ± 21.96d
		ST2	244.96 ± 0.23d	92.04 ± 0.39cde	734.7 ± 12.58fgh	652.1 ± 9.93de
	Filling stage	LT3	254.89 ± 0.15abc	99.29 ± 0.25a	836.2 ± 8.52a	756.8 ± 16.72a
		MT3	250.25 ± 0.80bcd	89.62 ± 0.42def	762.0 ± 23.38de	697.5 ± 6.83c
		ST3	245.34 ± 0.36d	85.42 ± 0.23fg	754.5 ± 9.80ef	668.1 ± 11.59d
		CK	253.74 ± 0.19abc	93.05 ± 0.31bcd	785.9 ± 12.58cd	705.2 ± 16.55c

T1, T2, and T3 refer to the different growth stages (booting stage, flowering stage and filling stage, respectively). CK, L, M and S refer to the different water treatments (flood-irrigated treatment, 0 kPa, light water stress treatment, -20 ± 5 kPa, moderate water stress treatment, -40 ± 5 kPa and severe water stress treatment, -60 ± 5 kPa, respectively). Lower case letters indicate that the contents of amylose and total starch of both cultivars are significantly different with the different treatments in the same column (P<0.05, LSD method). The data presented are the mean ± standard deviation, n = 3.

The highest amylose contents of both two cultivars were observed under LT2 while the lowest values were observed under ST3 in 2018. The two-year test results showed the same trend except that the highest amylose content of NJ 9108 was observed under LT3 in 2019. No significant differences were found among each light water stress treatment at different growth stages while amylose contents under LT2 were significantly higher than other treatments (except MT2 for NJ 9108). Compared with that under CK, the amylose content of IR72 increased by 1.13%, 2.07% and 0.59%, respectively, under LT1, LT2 and LT3, and decreased by 3.40%, 2.42% and 3.39%, respectively, under ST1, ST2 and ST3. The amylose content of NJ 9108 increased by -1.28%, 2.45% and 1.84%, respectively, under LT1, LT2 and LT3, and decreased by 6.83%, 4.91% and 8.26%, respectively, under ST1, ST2 and ST3 when compared with that under CK.

The highest total starch contents of both two cultivars were observed under LT3 while the lowest values were observed under ST1 in 2018. The two-year test results showed the same trend. No significant differences were found among each light water stress treatment at different growth stages while total starch contents under LT3 were significantly higher than other treatments. Compared with that under CK, the total starch content of IR72 increased by 2.76%, 2.91% and 3.44%, respectively, under LT1, LT2 and LT3, and decreased by 11.83%, 8.12% and 6.29%, respectively, under ST1, ST2 and ST3. The total starch content of NJ 9108 increased by 1.11%, 2.52% and 3.54%, respectively, under LT1, LT2 and LT3, and decreased by 12.49%, 8.87% and 5.86%, respectively, under ST1, ST2 and ST3 when compared with that under CK.

Taken together, these results indicated that light water stress could increase the contents of amylose and total starch in rice grains of which the effect at filling stage was more obvious. The effects of light water stress on amylose and total starch contents were more obvious at flowering stage and filling stage, respectively (except that the highest amylose content of NJ 9108 was obtained under LT3 in 2019). On the contrary, moderate and severe water stress could decrease the contents of amylose and total starch of which the effect at booting stage was more obvious. The effects of severe water stress on amylose and total starch contents were more obvious at booting stage (except that the lowest amylose content of NJ 9108 was obtained under ST3 in 2018).

#### 3.1.3 Starch synthesis-related enzyme activities

**Figure 5:**
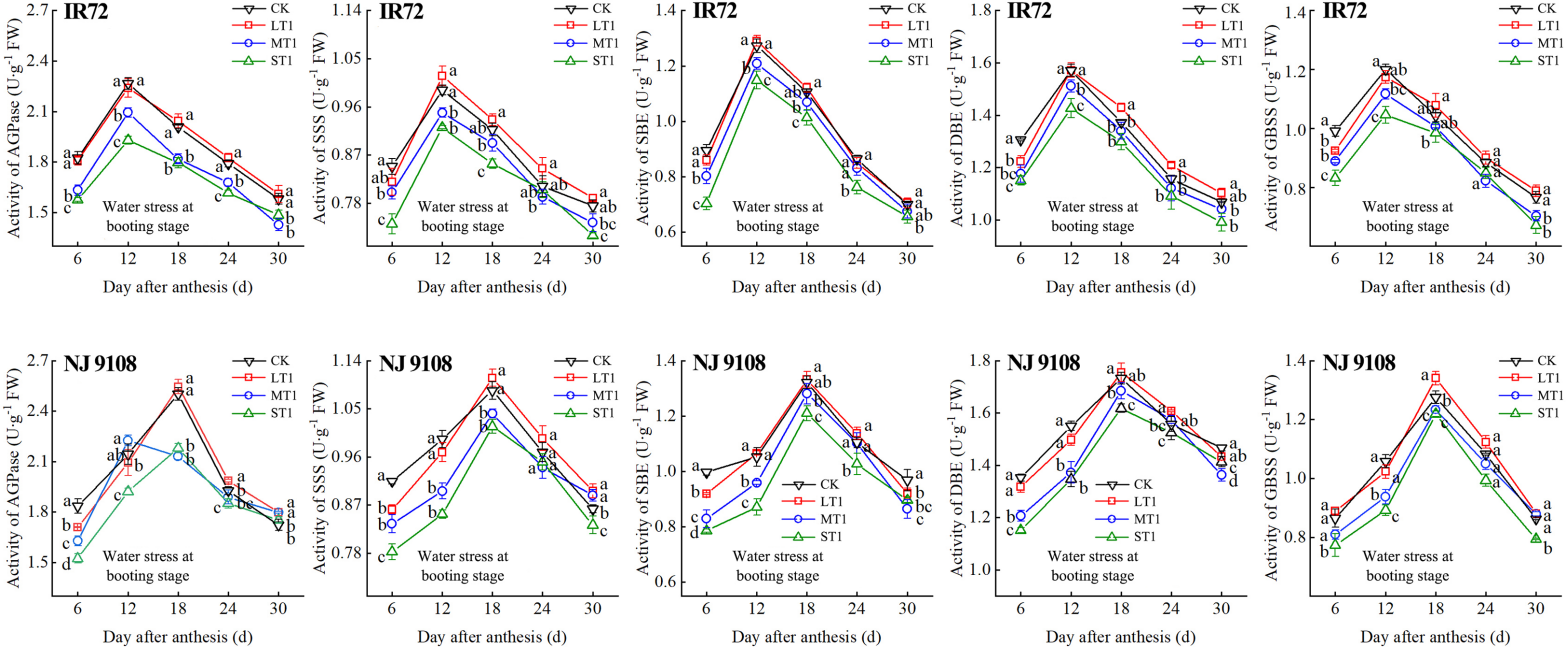
Effects of different water stress treatments on the activities of key enzyme involved in starch synthesis of IR72 and NJ 9108 at booting stage. T1 represents booting stage. CK, L, M and S refer to the different water treatments (flood-irrigated treatment, 0 kPa, light water stress treatment, -20 ± 5 kPa, moderate water stress treatment, -40 ± 5 kPa and severe water stress treatment, -60 ± 5 kPa, respectively). FW represents fresh weight. Lower case letters indicate that enzymes activities of both cultivars are significantly different with the different treatments (*P*<0.05, LSD method). The data presented are the mean ± standard deviation, *n* = 3.

**Figure 6:**
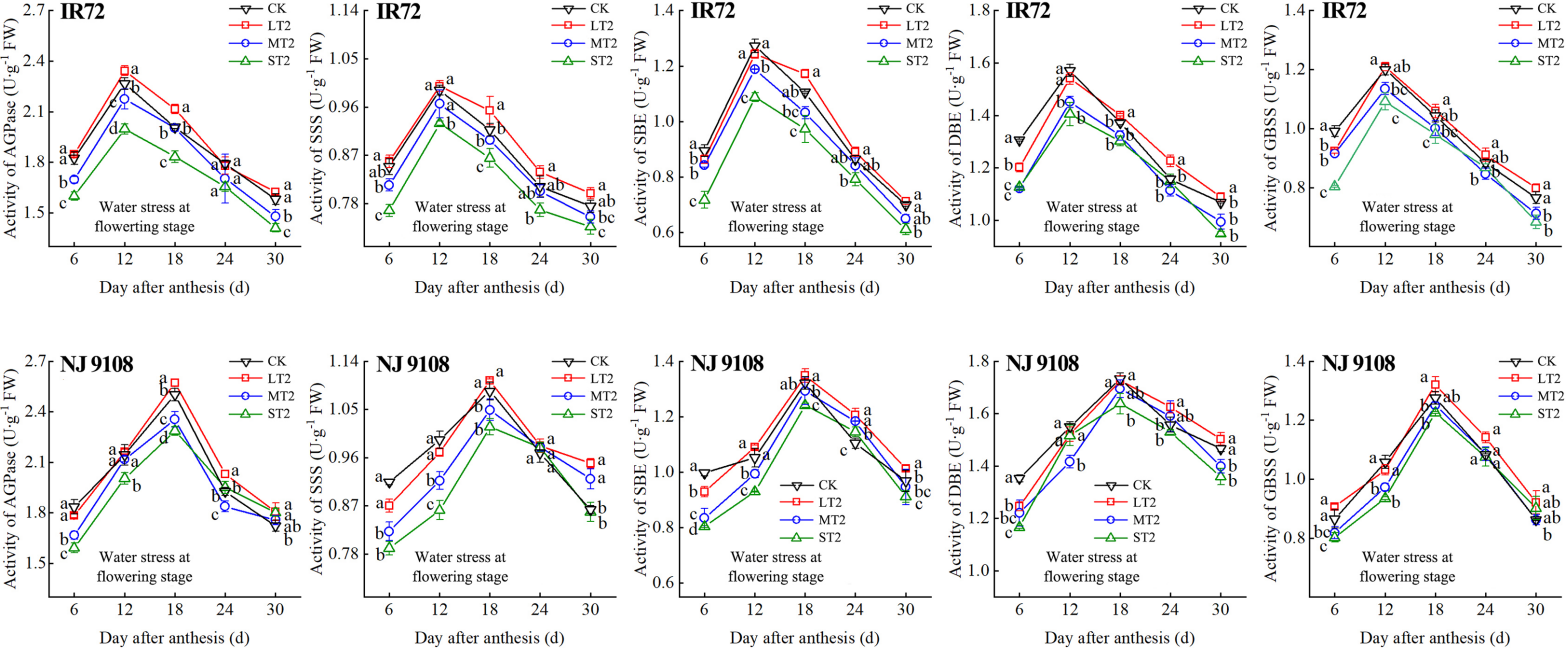
Effects of different water stress treatments on the activities of key enzyme involved in starch synthesis of IR72 and NJ 9108 at flowering stage. T2 represents flowering stage. CK, L, M and S refer to the different water treatments (flood-irrigated treatment, 0 kPa, light water stress treatment, -20 ± 5 kPa, moderate water stress treatment, -40 ± 5 kPa and severe water stress treatment, -60 ± 5 kPa, respectively). FW represents fresh weight. Lower case letters indicate that enzymes activities of both cultivars are significantly different with the different treatments (*P*<0.05, LSD method). The data presented are the mean ± standard deviation, *n* = 3.

**Figure 7:**
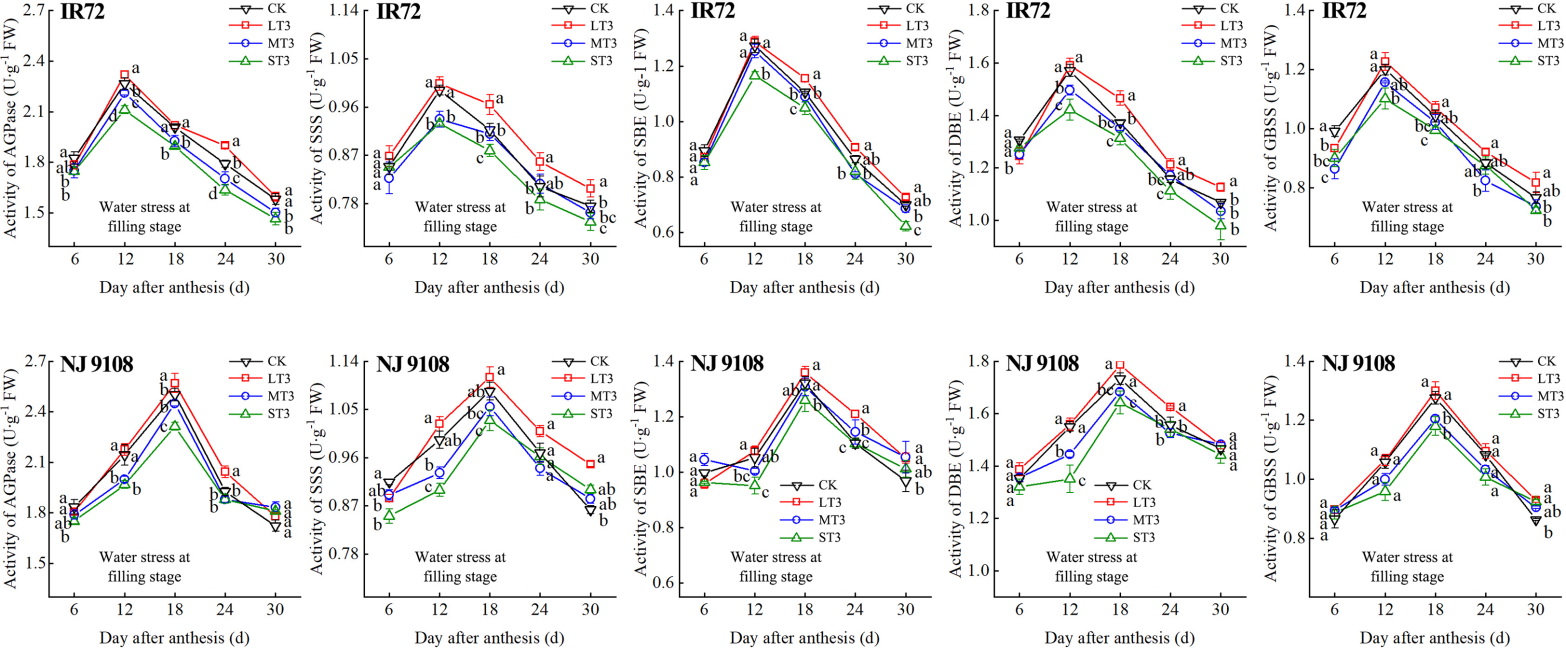
Effects of different water stress treatments on the activities of key enzyme involved in starch synthesis of IR72 and NJ 9108 at filling stage. T3 represents filling stage. CK, L, M and S refer to the different water treatments (flood-irrigated treatment, 0 kPa, light water stress treatment, -20 ± 5 kPa, moderate water stress treatment, -40 ± 5 kPa and severe water stress treatment, -60 ± 5 kPa, respectively). FW represents fresh weight. Lower case letters indicate that enzymes activities of both cultivars are significantly different with the different treatments (*P*<0.05, LSD method). The data presented are the mean ± standard deviation, *n* = 3.

During the grain filling process, the activities of AGPase, SSS, SBE, DBE and GBSS of both two cultivars first increased and then decreased (**Figures 5**–**7**). The peak activities of these enzymes of IR72 and NJ 9108 were obtained at 12 d and 18 d after flowering, respectively. The light water stress could increase the activities of starch synthesis-related enzyme while the moderate and severe water stress had the opposite effects. As the water stress increased, the activities of starch synthesis-related enzyme of both two cultivars under the same growth stage tended to be in the order of L > CK > M > S.

At 6 d after flowering, the highest activities of most starch synthesis-related enzyme were observed under CK while the lowest values were observed under S treatment (except GBSS of NJ 9108). The enzyme activities under CK and L at booting stage and flowering stage were higher than those under M and significantly higher than those under S, respectively (except DBE of IR72). At the peak stage, the enzyme activities of IR72 (12 d after flowering) and NJ 9108 (18 d after flowering) under CK and L were higher than those under M and significantly higher than those under S, respectively. Compared that under CK, the AGPase peak activities under LT1, LT2 and LT3 increased by -1.17%, 3.39% and 2.67% for IR72 and by 1.79%, 2.83% and 2.79% for NJ 9108, respectively, the SSS peak activities increased by 2.67%, 0.81% and 1.26% for IR72 and by 2.23%, 1.89% and 2.46% for NJ 9108, respectively, the SBE peak activities increased by 1.42%, -2.25% and 1.19% for IR72 and by 0.76%, 1.99% and 2.81% for NJ 9108, respectively, the DBE peak activities increased by -1.94%, -0.06% and 1.28% for IR72 and by 1.28%, -0.31% and 3.13% for NJ 9108, respectively, the GBSS peak activities increased by -2.15%, 0.85% and 2.27% for IR72 and by 5.13%, 3.61% and 1.95% for NJ 9108, respectively. At 24 d after flowering, the enzyme activities of both two cultivars decreased rapidly after the peak stage and the highest values were observed under L (except SBE of IR72 at booting stage). At 30 d after flowering, the enzyme activities of both two cultivars under LT1 were lower than those under LT2 and LT3 (except AGPase). However, the enzyme activities under L at different growth stages were higher than those under M and S.

#### 3.1.4 Expression analysis of *OsSuS* family and *OsVIN3*

**Figure 8:**
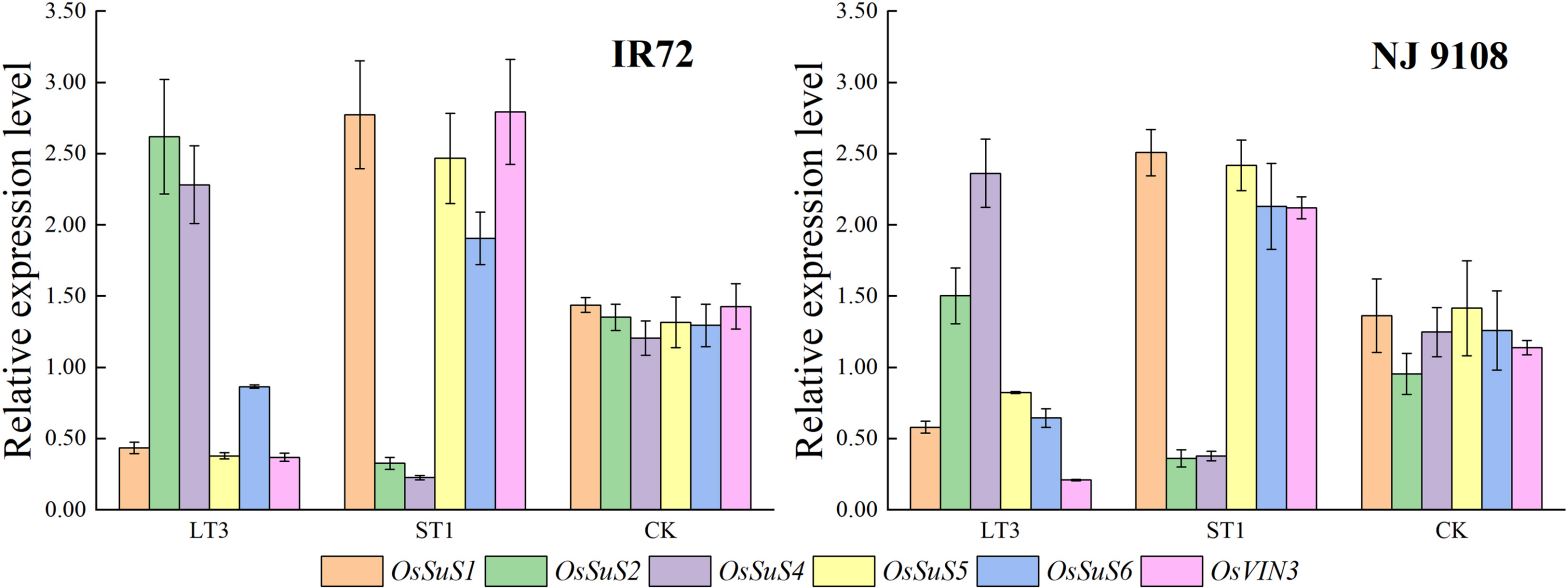
Effects of different water stress treatments on expression levels of *OsSuS* family and *OsVIN3* in rice grains at different growth stages. LT3, ST1 and CK refer to light water stress treatment at filling stage (-20 ± 5 kPa), severe water stress treatment at booting stage (-60 ± 5 kPa) and flood-irrigated treatment (0 kPa), respectively. The data presented are the mean ± standard deviation, *n* = 3.

According to the above results, the peak activities of starch synthesis-related enzymes of IR72 and NJ 9108 were obtained at 12 d and 18 d after flowering, respectively. And the highest starch contents of both two cultivars were obtained under LT3 while the lowest were obtained under ST1. Therefore, samples from these two different growth stages and water treatments were selected for expression analysis of *OsSuS* family and *OsVIN3*. The results showed that *OsSuS2* and *OsSuS4* had high expression levels under LT3 (**Figure 8**). Despite the expression levels of *OsSuS1*, *OsSuS5*, *OsSuS6* and *OsVIN3* were high under ST1, starch content in rice grain under ST1 was lower than LT3, indicating that these genes might play minor role in the increased capacity for starch synthesis.

### 3.2 Yield and yield components

No obvious impacts were observed on productive panicle number per pot and filled grain number per panicle between different water stress treatments. However, as the water stress increased, the seed setting rate, 1000-grain weight and grain yield of both two cultivars under the same growth stage decreased significantly. The two-year test results showed that the highest values were obtained under LT3 and the lowest values were obtained under ST1 (**Tables 5**, **6**).

**Table 5:** Effects of different water stress treatments on yield and yield components of IR72 at different growth stages.

Year	Growth stage	Treatment	PPN (number pot^-1^)	FGN (number panicle^-1^)	SSR (%)	1000-GW (g)	GY (g pot^-1^)
2018	Booting stage	LT1	24.02 ± 1.11c	88.58 ± 2.73c	73.60 ± 0.11de	28.08 ± 0.14c	62.96 ± 2.18d
		MT1	29.07 ± 0.85ab	78.37 ± 5.83d	60.81 ± 1.48h	24.01 ± 0.09i	54.16 ± 0.85fg
		ST1	29.94 ± 0.87a	78.59 ± 5.73d	58.27 ± 1.50i	23.59 ± 0.08j	49.14 ± 2.02g
	Flowering stage	LT2	27.24 ± 1.51abc	101.89 ± 4.33a	76.20 ± 0.79c	28.52 ± 0.29b	69.70 ± 4.54b
		MT2	24.64 ± 1.36bc	100.78 ± 4.52ab	65.92 ± 1.33f	25.42 ± 0.25g	59.21 ± 5.34def
		ST2	25.38 ± 1.49abc	100.52 ± 4.38ab	63.51 ± 1.37g	24.45 ± 0.12h	55.89 ± 0.66ef
	Filling stage	LT3	25.74 ± 1.30abc	107.33 ± 4.39a	81.11 ± 0.39a	28.92 ± 0.15a	77.03 ± 5.77a
		MT3	23.32 ± 1.53c	93.88 ± 1.49bc	74.43 ± 1.06cd	26.61 ± 0.11e	63.67 ± 2.32cd
		ST3	23.92 ± 1.57c	92.78 ± 1.41c	72.37 ± 1.12e	25.99 ± 0.07f	60.97 ± 1.71de
		CK	26.35 ± 1.20abc	102.72 ± 5.32a	78.75 ± 0.13b	27.64 ± 0.03d	69.03 ± 0.86bc
2019	Booting stage	LT1	27.75 ± 0.51a	110.90 ± 1.51cd	72.85 ± 1.29c	27.27 ± 0.17b	43.51 ± 3.48b
		MT1	28.72 ± 1.13a	114.82 ± 2.08abc	68.42 ± 0.95d	21.95 ± 0.24i	30.42 ± 2.60c
		ST1	27.36 ± 1.10a	116.82 ± 4.82ab	65.89 ± 1.92e	21.52 ± 0.23j	29.51 ± 2.52c
	Flowering stage	LT2	27.15 ± 1.19a	117.35 ± 1.32a	76.70 ± 1.23b	26.22 ± 0.22c	55.47 ± 4.45a
		MT2	28.38 ± 1.89a	111.06 ± 2.07cd	73.81 ± 0.34c	23.81 ± 0.10g	38.72 ± 4.61b
		ST2	28.11 ± 0.96a	117.22 ± 2.40a	69.41 ± 0.44d	23.34 ± 0.10h	37.56 ± 4.47b
	Filling stage	LT3	27.36 ± 0.78a	119.68 ± 5.53a	81.27 ± 0.46a	27.80 ± 0.02a	60.84 ± 5.05a
		MT3	28.96 ± 0.76a	108.22 ± 2.37d	76.35 ± 1.37b	24.61 ± 0.08e	39.90 ± 1.94b
		ST3	28.60 ± 0.95a	111.76 ± 0.56cd	73.43 ± 0.68c	24.11 ± 0.08f	38.70 ± 1.88b
		CK	27.85 ± 1.24a	111.88 ± 2.74bcd	80.23 ± 0.90a	25.81 ± 0.10d	54.87 ± 2.07a

PPN, FGN, SSR, 1000-GW and GY represent the productive panicle number per pot, filled grain number per panicle, seed setting rate, 1000-grain weight and grain yield per pot, respectively. T1, T2, and T3 refer to the different growth stages (booting stage, flowering stage and filling stage, respectively). CK, L, M and S refer to the different water treatments (flood-irrigated treatment, 0 kPa, light water stress treatment, -20 ± 5 kPa, moderate water stress treatment, -40 ± 5 kPa and severe water stress treatment, -60 ± 5 kPa, respectively). Lower case letters indicate that the yield and yield components of both cultivars are significantly different with the different treatments in the same column (P<0.05, LSD method). The data presented are the mean ± standard deviation, n = 3.

**Table 6:** Effects of different water stress treatments on yield and yield components of NJ 9108 at different growth stages.

Year	Growth stage	Treatment	PPN (number pot^-1^)	FGN (number panicle^-1^)	SSR (%)	1000-GW (g)	GY (g pot^-1^)
2018	Booting stage	LT1	18.76 ± 0.38ab	136.61 ± 2.59c	75.12 ± 0.91bc	22.50 ± 0.19c	41.43 ± 0.45cd
		MT1	19.40 ± 0.86ab	124.71 ± 1.86d	68.47 ± 1.33e	18.01 ± 0.21g	22.62 ± 1.23g
		ST1	19.99 ± 0.89a	124.04 ± 1.84d	66.15 ± 1.38f	17.60 ± 0.06h	21.94 ± 1.19g
	Flowering stage	LT2	19.67 ± 0.40ab	147.50 ± 1.88ab	76.09 ± 0.99b	22.79 ± 0.11b	45.68 ± 0.62b
		MT2	19.46 ± 0.76ab	152.99 ± 2.37a	70.79 ± 0.65d	20.74 ± 0.08e	33.11 ± 1.27f
		ST2	20.04 ± 0.0.78a	151.80 ± 2.45a	68.56 ± 0.67e	20.02 ± 0.10f	32.12 ± 1.23f
	Filling stage	LT3	18.47 ± 0.12b	147.33 ± 4.51ab	78.90 ± 0.79a	23.82 ± 0.14a	49.71 ± 1.67a
		MT3	18.57 ± 0.48b	146.97 ± 5.92ab	74.18 ± 1.13c	20.95 ± 0.08e	38.89 ± 2.89de
		ST3	19.13 ± 0.49ab	145.29 ± 5.69b	72.11 ± 1.18d	20.23 ± 0.19f	37.72 ± 2.80e
		CK	18.87 ± 1.52ab	147.36 ± 4.96ab	78.76 ± 0.37a	21.79 ± 0.09d	42.85 ± 2.20bc
2019	Booting stage	LT1	18.20 ± 0.30abc	141.27 ± 4.16c	73.63 ± 1.00cd	21.52 ± 0.10b	42.60 ± 1.52cd
		MT1	18.61 ± 0.61ab	150.68 ± 5.77ab	71.86 ± 0.08e	13.65 ± 0.21i	36.26 ± 1.42e
		ST1	19.37 ± 0.77a	153.97 ± 4.63a	68.23 ± 0.22f	13.38 ± 0.20j	35.85 ± 2.35e
	Flowering stage	LT2	19.11 ± 0.45ab	148.56 ± 1.52abc	77.44 ± 0.79b	20.70 ± 0.16c	50.11 ± 1.19ab
		MT2	18.02 ± 0.37bc	149.49 ± 0.28abc	74.29 ± 0.42c	15.72 ± 0.09g	41.50 ± 0.50d
		ST2	18.31 ± 0.51abc	150.98 ± 2.88ab	72.27 ± 0.70de	15.40 ± 0.09h	39.99 ± 0.24de
	Filling stage	LT3	18.14 ± 1.00bc	147.56 ± 4.07abc	83.73 ± 1.45a	23.16 ± 0.18a	53.44 ± 4.66a
		MT3	17.95 ± 0.65bc	145.10 ± 5.28bc	77.87 ± 0.86b	19.19 ± 0.06e	42.59 ± 4.80cd
		ST3	17.26 ± 1.25c	145.55 ± 5.83abc	76.98 ± 1.59b	18.81 ± 0.06f	39.23 ± 4.74de
		CK	18.22 ± 0.45abc	142.49 ± 3.04bc	82.27 ± 0.29a	19.60 ± 0.13d	46.52 ± 0.45bc

PPN, FGN, SSR, 1000-GW and GY represent the productive panicle number per pot, filled grain number per panicle, seed setting rate, 1000-grain weight and grain yield per pot, respectively. T1, T2, and T3 refer to the different growth stages (booting stage, flowering stage and filling stage, respectively). CK, L, M and S refer to the different water treatments (flood-irrigated treatment, 0 kPa, light water stress treatment, -20 ± 5 kPa, moderate water stress treatment, -40 ± 5 kPa and severe water stress treatment, -60 ± 5 kPa, respectively). Lower case letters indicate that the yield and yield components of both cultivars are significantly different with the different treatments in the same column (P<0.05, LSD method). The data presented are the mean ± standard deviation, n = 3.

In 2018, the seed setting rate, 1000-grain weight and grain yield of both two cultivars under LT3 were significantly higher than other treatments. Compared with that under CK, the seed setting rate increased by -5.15, -2.55 and 2.36 percentage points for IR72 and by -3.64, -2.67 and 0.14 percentage points for NJ 9108, respectively, under LT1, LT2 and LT3, while decreased by 20.48, 15.24 and 6.38 percentage points for IR72 and by 12.61, 10.20 and 6.65 percentage points for NJ 9108, respectively, under ST1, ST2 and ST3. The 1000-grain weight increased by 1.59%, 3.18% and 4.63% for IR72 and by 3.26%, 4.59% and 9.32% for NJ 9108, respectively, under LT1, LT2 and LT3, while decreased by 14.65%, 11.54% and 5.97% for IR72 and by 19.23%, 8.12% and 7.16% for NJ 9108, respectively, under ST1, ST2 and ST3. The grain yield increased by -8.79%, 0.97% and 11.59% for IR72 and by -3.31%, 6.60% and 16.01% for NJ 9108, respectively, under LT1, LT2 and LT3, while decreased by 28.81%, 19.04% and 11.68% for IR72 and by 48.80%, 25.04% and 11.97% for NJ 9108, respectively, under ST1, ST2 and ST3. The two-year test results showed the same trend.

Taken together, these results indicated that light water stress could increase the 1000-grain weight at different growth stages. The seed setting rate could be enhanced under LT3 while the grain yield could be enhanced under LT2 and LT3. The 1000-grain weight and grain yield of both two cultivars under the same water treatments tended to be in the order of T3 > T2 > T1.

### 3.3 Principle component analysis

To sum up, the responses of starch synthesis and accumulation and yield of two rice cultivars to water treatments at different growth stages were different. The starch content and rice yield consists of many evaluation indexes and a single index could not objectively reflect the effects of water stress on them. PCA is a simple and effective statistical tool that is widely used in dimensionality reduction and factorial analysis of high-dimension datasets. Datasets with several correlated variables are decomposed into a smaller number of linearly independent variables by PCA. Hence, PCA of 10 indexes including five starch synthesis-related enzymes activities, amylose and total starch contents, seed setting rate, 1000-grain weight and yield of two rice cultivars under different water treatments was used to establish a comprehensive evaluation model (**Table 7**).

**Table 7:** The comprehensive scores and rankings of IR72 and NJ 9108 under different water treatments.

Growth stage	Treatment	Cultivar			
		IR72		NJ 9108	
		Score	Ranking	Score	Ranking
Booting stage	LT1	-0.57	3	1.21	3
	MT1	-1.88	8	-0.01	8
	ST1	-2.43	10	-0.66	9
Flowering stage	LT2	-0.33	2	1.62	2
	MT2	-1.53	6	0.43	6
	ST2	-2.34	9	-0.01	8
Filling stage	LT3	-0.12	1	1.81	1
	MT3	-1.32	5	0.78	5
	ST3	-1.68	7	0.39	7
	CK	-0.66	4	1.17	4

T1, T2, and T3 refer to the different growth stages (booting stage, flowering stage and filling stage, respectively). CK, L, M and S refer to the different water treatments (flood-irrigated treatment, 0 kPa, light water stress treatment, -20 ± 5 kPa, moderate water stress treatment, -40 ± 5 kPa and severe water stress treatment, -60 ± 5 kPa, respectively).

The comprehensive evaluation results showed that LT3 got the highest comprehensive score while ST1 got the lowest, and the comprehensive scores of both LT1 and LT2 were higher than CK. As the water stress increased, the comprehensive scores of both two cultivars decreased and tended to be in the order of NJ 9108 > IR72.

Taken together, these results indicated that light water stress could promote starch synthesis and accumulation and increase rice yield while moderate and severe water stress had the opposite effects. The effects of improving starch content and rice yield under light water stress treatment at different growth stages followed the trend of T3 > T2 > T1. And the drought resistance ability of NJ 9108 was better than IR72 according to the comprehensive scores of PCA.

## 4 Discussion

### 4.1 Effects of water stress on starch synthesis-related enzyme activities

Photosynthates transported into grain mainly exist in the form of sucrose through the phloem at first, and then stored as starch through a series of enzymatic reactions. The key enzyme activities involved in sucrose-to-starch conversion in rice grain determine the sugar content, starch synthesis and accumulation, rice yield and quality (Zhang et al., 2012). Two types of enzymes are responsible for the degradation of sucrose: SuS and invertase (INV). SuS reversibly catalyzes the hydrolysis of sucrose into UDP-glucose and fructose (Coleman et al., 2009). In contrast, INV degrades sucrose into glucose and fructose irreversibly (Ruan, 2014). Based on subcellular location, INVs are classified into cell wall invertase (CWIN), vacuolar invertase (VIN) and cytoplasmic invertase (CIN) (Roitsch and Gonzalez, 2004). VIN regulates cell expansion, osmotic pressure, sugar signals, sucrose accumulation, and sucrose concentration, especially during the expansion phases of sink organs (Li et al., 2017). It is reported that appropriate water stress could increase the activities and expression level of SuS and VIN (Zhang et al., 2017; Wang et al., 2019). SuS activity was substantially enhanced by water stress, and was positively correlated with starch accumulation rate in the grains (Yang et al., 2003). VIN activity increased significantly, and the more severe the drought, the higher the VIN activity (Wang et al., 2022). We observed the same results in the present study that the expression levels of *OsSuS2* and *OsSuS4* increased under light water stress treatment (Wang et al., 2015a) and *OsVIN3* expression level under severe water stress treatment was higher than that under light water stress treatment (Wang, 2020). However, reports about the effects of water stress on INV activities and expression levels in rice grains are still limited. Changes in the expression level of *OsVIN3* were not consistent with those in the starch content under severe water stress treatment. Further research is needed to identify the role of *OsCWIN* and *OsVIN* family genes in sucrose-to-starch conversion in rice grains and their regulatory factors when subjected to water stress during grain filling.

The immediate precursor for the starch synthesis in rice grain is adenosine diphosphate glucose (ADPG) which is synthesized from glucose-1-phosphate and ATP, and the reaction is catalyzed by AGPase (Kawagoe et al., 2005). SSS and GBSS are involved in amylopectin and amylose synthesis, respectively, which both utilize ADPG as the substrate (Akihiro et al., 2005; Szydlowski et al., 2011). SBE catalyzes the formation of α-1,6-glycoside bond, while DBE hydrolyzes α-1,6-glycoside bond (Nakamura et al., 2010). Previous studies have found that water stress during rice filling stage will reduce the starch synthesis-related enzyme activities (Shi et al., 2016; V et al., 2019). However, some studies have reported that a moderate wetting drying regime during the grain-filling stage of rice can increase the activities of key enzymes involved in sucrose-to-starch metabolic pathway (Zhang et al., 2008). In the present study, we observed that light water stress at different growth stages all increased the activities of AGPase, SSS, GBSS, SBE and DBE. The moderate and severe water stress had the opposite effects. With the increasing level of water stress, the key enzyme activities decreased, in agreement with the results of previous studies (Zhang et al., 2008). The activities and peak activities of these five enzymes during mid and late filling stage increased under light water stress at different growth stages. This might be due to the fact that light water stress promote the roots developmentand improve the plants vitality, resulting in an increase in sucrose-to-starch synthesis-related enzyme activity.

### 4.2 Effects of water stress on starch synthesis and accumulation

Photosynthates are primarily transported in the form of sucrose from source to sink tissues (Pandey and Shukla, 2015). The transport and distribution of total soluble sugar and sucrose are key processes in plants response to abiotic stresses (Mathan et al., 2021). Previous studies have found that the increase in sucrose content under the drought in the developing grains could be due to the reduction in the activity of the sucrose synthase which is the main enzyme involved in the breakdown of sucrose (V et al., 2019). Starch is the major component of rice. Starch composition and structure are closely correlated with rice yield and quality (Li et al., 2018). Total soluble sugar, sucrose and starch can be converted into each other, and their content determines the accumulation of starch in rice grain (Wang and Zhang, 2020). Moderate drought stress during grain filling (water deficit stress was initiated by withholding irrigation during the booting initiation stage and continued for 21 days) accelerates the transport of photosynthate stored in vegetative organs to grains leading to increased starch (V and Tyagi, 2020). In the present study, we observed that total starch content under light water stress at different growth stages was higher than that under CK while total soluble sugar and sucrose contents were lower than that under CK. And the moderate and severe water stress showed the opposite trend. These results indicated that light water stress promoted the conversion of total soluble sugar and sucrose to starch through regulating the key enzymes involved in starch synthesis, and increased the starch content in rice grain as a result.

### 4.3 Effects of water stress on rice yield and yield components

Rice yield, in response to drought, depends on the timing of the drought event in relation to plant growth stage. As the water stress level and timing increase, rice yield usually decreases significantly especially during rice water sensitive stage (Farooq et al., 2009; Rao et al., 2019). Drought stress at booting stage inhibites the development of branches and spikelets leading to spike degeneration and pollen abortion which have significant impact on grain number and seed setting rate (Dolferus et al., 2011). When the rice is exposed to severe drought stress at flowering stage, the reduction of spikelets per panicle, filled grains and grain yield are observed due to the significant increase in spikelets sterility (Yang et al., 2019). Drought stress at filling stage enhances the plant senescence and remobilization of carbon store from leaves and stems to grains. The reduction of yield could be due to a decrease in the grain filling period under the drought at filling stage (Yang et al., 2008). The results of this study showed that water stress at booting stage was not favorable to grain yield. On the contrary, light water stress at flowering stage and filling stage could increase rice yield which followed the trend of filling stage > flowering stage. The seed setting rate increased only under light water stress at filling stage while the 1000-grain weight increased under light water stress at different growth stages. However, both moderate and severe water stress decreased 1000-grain weight. With the increasing level of water stress at the same growth stage, the 1000-grain weight and grain yield decreased significantly, in agreement with the results of previous studies (Ye et al., 2013). The increase in 1000-grain weight under LT1 might due to the compensation effect caused by soil water condition after re-watering. Additionally, the decrease of effective panicle number might not be the main factor of rice yield reduction since the water stress treatment was applied after the number of rice tillers reached the peak. A considerable number of aborted pollen limiting seed setting rate under a soil water deficit at booting stage was a major constraint for rice yield (Kato et al., 2008).

## 5 Conlusion

Water is an important factor in agricultural and food production, while water stress impairs rice yield and quality, thus being a severe threat to sustainable agriculture. The starch content is one of the main factors affecting rice yield and quality but little information is available on starch accumulation and related enzyme activities under different water stress treatments at different growth stages. Our study showed that light water stress at filling stage was beneficial to water-saving and rice yield improvement. Different water stress treatments at booting stage all led to decreases in rice yield. The sucrose content and total soluble sugar content increased, while starch synthesis-related enzyme activities, amylose content, total starch content, seed setting rate, 1000-grain weight and yield decreased with the increasing level of water stress at the same growth stage. The PCA results showed that light water stress could promote starch synthesis and accumulation and increase rice yield, and the effects of improving starch content and rice yield at different growth stages tended to be in order of T3 > T2 > T1. The grain yield under LT3 increased by 11.59% for IR72 and 16.01% for NJ 9108, respectively when compared with CK. Therefore, we suggested that light water stress could be applied at filling stage to increase rice yield in the practical production. This new water management may offer a beneficial option for farmers to save water and labor force and maintain high yield of rice.

## Data availability statement

The original contributions presented in the study are included in the article/**Supplementary Material**. Further inquiries can be directed to the corresponding authors.

## Author contributions

All authors listed have made a substantial, direct, and intellectual contribution to the work and approved it for publication.
