# Supplementary material for: Effects of water stress on starch synthesis and accumulation of two rice cultivars at different growth stages
Source: Front Plant Sci. 2023 Apr 25;14:1133524. doi: 10.3389/fpls.2023.1133524 (PMC10166795; doi:10.3389/fpls.2023.1133524)
Supplement: Supplementary file 1 [file Table_1.doc]

Supplementary Table 1 Gene primers used for PCR amplification

| Gene ID | Forward primers (5'-3') | Reverse primers (5'-3') |
| --- | --- | --- |
| OsSuS1 | CATCTCAGGCTGAGACTCTGA | CAAATTCAATCGACCTTACTT |
| OsSuS2 | GAGGCTGATGACCTTGACTGG | CCCTCCATTACTTGGATGTGCT |
| OsSuS4 | TGAACTGGCGAAGACTGTACC | CAATGGCAATGAAGCACTGAG |
| OsSuS5 | CAGGTTAGCCGGGCAGAAG | CATTGTACACAAATCTCACGCAAG |
| OsSuS6 | CGAACAACCAGCACAACCTAC | GGAGCAGTGGTCCAAGTAAG |
| OSINV3 | TCTTGCTGGCCTGGGTTGTTTG | ATTTGCCACGTCCCAGGCTTTG |
| OsUBQ1 | CAGTAAGTCCTCAGCCAT | AGACCAGACAACCATAGC |
